# Harmonisation of neuropsychological assessment for Alzheimer’s disease diagnosis across diverse African settings: A Pilot Study from the African Dementia Consortium (AfDC)

**DOI:** 10.1002/alz.093348

**Published:** 2025-01-09

**Authors:** Stella‐Maria Paddick, Michael L. Cuccaro, Andrea Damas, Olusegun Baiyewu, Temitope Hannah Farombi, Olufisayo Oluyinka Elugbadebo, Njideka U. Okubadejo, Oluwadamilola Ojo, Andrew F. Zaman, Katalina F. McInerney, Albert Kwaku Akpalu, Ruth Laryea, Fred Stephen Sarfo, Shadrack Osei, Reginald Obiako, David Ndetei, Christine W. Musyimi, Albertino Damasceno, Thierry Adoukonou, Biniyam A. Ayele, Pedro R. Mena, Richard Walker, Rajesh N Kalaria, Jeffery M. Vance, Adesola Ogunniyi, Margaret A. Pericak‐Vance, Rufus O. Akinyemi, African Dementia Consortium

**Affiliations:** ^1^ Newcastle University, Newcastle, Newcastle Upon Tyne United Kingdom; ^2^ John P. Hussman Institute for Human Genomics, University of Miami Miller School of Medicine, Miami, FL USA; ^3^ Dodoma Neuropsychiatric Hospital, Dodoma Tanzania, United Republic of; ^4^ University College Hospital, Ibadan, Oyo State Nigeria; ^5^ University College Hospital, Ibadan Nigeria; ^6^ College of Medicine, University of Ibadan, Ibadan Nigeria; ^7^ African Dementia Consortium, Lagos Nigeria; ^8^ College of Medicine, University of Lagos, Lagos Nigeria; ^9^ University of Miami Miller School of Medicine, Miami, FL USA; ^10^ University of Ghana, Accra Ghana; ^11^ Komfo Anokye Teaching Hospital, Kumasi Ghana; ^12^ Ahmadu Bello University, Zaria, Kaduna Nigeria; ^13^ Africa Mental Health Research and Training Foundation, Nairobi Kenya; ^14^ University of Nairobi, Nairobi Kenya; ^15^ Eduardo Mondlane University, Maputo Mozambique; ^16^ University Teaching Hospital, Department of Neurology,, Parakou Benin; ^17^ Addis Ababa University, Addis Ababa, Addis Ababa Ethiopia; ^18^ Newcastle University, Newcastle United Kingdom; ^19^ John P. Hussman Institute for Human Genomics, Miami, FL USA; ^20^ College of Medicine, University of Ibadan, Ibadan, Oyo State Nigeria; ^21^ Neuroscience and Aging Research Unit, Institute of Advanced Medical Research and Training, College of Medicine, University of Ibadan, Ibadan Nigeria; ^22^ Institute for Advanced Medical Research and Training, College of Medicine, University of Ibadan, Ibadan, Oyo State Nigeria

## Abstract

**Background:**

Majority of people living worldwide live in low‐ and middle‐ income countries, including sub‐Saharan Africa (SSA). Most cognitive assessment batteries for Alzheimer’s Disease(AD), are developed in high income countries (HICs), where most international dementia collaborations and data originate. The African Dementia Consortium (AfDC) is a new scientific collaboration network currently participating in the Recruitment and Retention for Alzheimer’s Disease Diversity Genetic Cohorts in the Alzheimer’s Disease Sequencing Project (READD‐ADSP). This pilot study investigated essential adaptations and pilot of cognitive measures from the US Uniform Data Set Version 3 (UDS3) alongside SSA specific measures where these exist, in diverse cultural contexts of the AfDC.

**Method:**

A minimum of 24 individuals (12 with >12 years education, 12 with < 12 years education) aged over 60 years were recruited in Nigeria (3 sites), Tanzania, Kenya, Ghana, Mozambique, Benin and Ethiopia, using convenience sampling. Tests were administered according to a culturally adapted protocol. Feasibility data included time taken for completion, proportion of ‘complete failure’ on individual items, language and translation challenges.

**Result:**

Data were available from 10 sites across 7 countries. Mean scores on some US measures (MINT, Benson figure copy, word list recall) with minor adaptations, were similar to US norms. In contrast, other tests appeared highly challenging (e.g., Trail Making Test, Craft Story) with median scores well outside the HIC range and a high rate of ‘total failure’ (Trails B). Cultural adaptations were necessary and unexpectedly challenging including sound selection and categorical conceptualisation for verbal fluency tasks and protocolising for bilingualism. Median scores varied by site and education, highlighting the need for appropriate local normative values. All adaptations and modifications were carefully documented and will be used by the ADSP‐Phenotype Harmonization Consortium to integrate AfDC cognitive test data with other ADSP cohort data.

**Conclusion:**

Further work should outline required steps for cross cultural harmonisation across countries, especially in SSA as a guideline for other collaborations. The publication of normative values for frequently used AD cognitive measures specific to diverse SSA sociocultural contexts of SSA is needed.

